# Effects of a work-related stress model based mental health promotion program on job stress, stress reactions and coping profiles of women workers: a control groups study

**DOI:** 10.1186/s12889-020-09769-0

**Published:** 2020-11-04

**Authors:** Ozlem Koseoglu Ornek, Melek Nihal Esin

**Affiliations:** 1 Occupational and Environmental Epidemiology & NetTeaching Unit, Institute and Clinic for Occupational, Social and Environmental Medicine, University Hospital, LMU, Ziemssenstr. 1, 80336 Munich, Germany; 2 Department of Nursing, Faculty of Health Sciences, Istanbul Bilgi University, Dolapdere Kampus, Hacıahmet Mahallesi, Pir Hüsamettin Sokak No: 20, 34440 Beyoğlu/ Istanbul, Turkey; 3 Department of Public Health Nursing, İstanbul University-Cerrahpasa Florence Nightingale Faculty of Nursing, İstanbul Üniversitesi Florence Nightingale Hemşirelik Fakültesi, Abide-i Hürriyet Caddesi, Şişli/İstanbul, Turkey

**Keywords:** Occupational stress, Work related stress model, Job stress, Women workers, Coping profiles, Cortisol, Immunoglobulin A

## Abstract

**Background:**

Work-related stress and its detrimental effects on human health have rapidly increased during the past several years. It causes many different stress reactions, related diseases and unhealthy behavior among workers, but especially women workers. Thus, the aim of this study was to examine the effects of the work-related stress model based Workplace Mental Health Promotion Programme on the job stress, social support, reactions, salivary immunoglobulin A and Cortisol levels, work absenteeism, job performance and coping profiles of women workers.

**Methods:**

This study had a “pre-test post-test non-equivalent control groups” design and included 70 women workers (35 in each study group) selected by randomized sampling from two factories. The programme was delivered as an intervention including 12 weeks of follow-up. Reminder messages, videos, and WhatsApp texts were used at the follow-up stage. The research measurements were; the assessment form, the Brief Job Stress Questionnaire, the Brief Coping Profile Scale, salivary ELISA kits, and a self-reported check-list.

**Results:**

There were no differences in sociodemographic characteristics, general health or working conditions between the Intervention and control groups(*p* > .05). Three months after the intervention, there was a significant decrease in job stress(*p* ≤ .001), physical and mental reactions’ scores(*p* ≤ .001) and work absenteeism(*p* < .05), and there was an increase in job performance(*p* < .05), social support(*p* ≤ .001) among the intervention group. The programme showed positive effects on coping profiles(*p* < .05). After the intervention salivary-cortisol and IgA levels showed a statistically significant decrease(*p* < .05). A majority of effect sizes were very large (η_p_^2^ > .14).

**Conclusions:**

Work-ProMentH was found to be effective and useful in job stress management and promotion of effective coping profiles. It enables its users to holistically assess worker stress and to plan and examine intervention programmes via a systematic approach. There is a need for more empirical studies that may support the data of the present study, but it is thought that the intervention can be maintained for the long-term. We recommend that occupational health professionals at workplaces should consider using this model-based cost-effective intervention, which seems easy and practical to apply in real-life situations.

**Trial registration:**

ISRCTN registration ID: ISRCTN14333710 (2020/10/03, retrospective registration).

## Background

Work-related stress (WRS) has become a crucial public health problem in recent decades, and its detrimental effects on human health have recently increased rapidly [1, 2]. Thus, there is a large challenge to understand its reactions, related factors and outcomes. Many stress-related models have been developed to better explain and cope with the stress [3–7]. According to the WRS model, stress is defined as all reactions that take place and cause any change in individuals’ cognitive, physical, psychological and emotional structures as a result of a high perceived workload [6]. It has a flow process, and causes many different stress reactions, related diseases and unhealthy behaviours among workers. The reactions can involve physical, psychological, biological, and/or behavioural symptoms. Commonly observed physical symptoms include high blood pressure, a fast pulse, Cheyne Stokes respiration, headache, and tense muscles. Biological parameters consist mainly of immunological variables, such as T cell activation, decreased immunoglobulin A (IgA), and increased cortisol secretion [8–10]. Mental health symptoms may involve irritability, tension, aggressive behaviors, lack of concentration, and sleep, perception, and memory disorders [11, 12]. If the reactions persist for an extended period, there may be irreversible health outcomes, such as chronic fatigue, cardiovascular diseases [13, 14], musculoskeletal diseases [15], or mental health problems, such as anxiety or depression [16, 17]. The development of such physical and mental health problems can also lead to extended sick leaves or absenteeism [18, 19] and decreases in job quality, performance, and productivity; it can also threaten workers’ health and safety [6, 20].

**Fig. 1:**
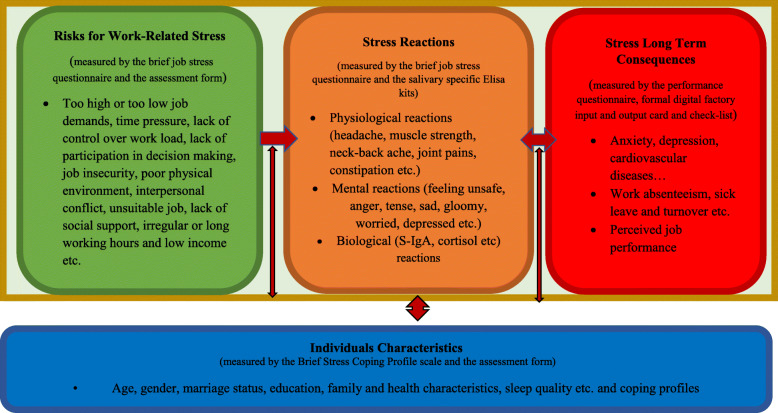
The evaluation of the Model of Work-related Stress’ components and their interrelations

Working conditions and individual characteristics are the main related factors for developing WRS. The work-related stress model indicates that, stress and its reactions occur as a result of the relationship between individual characteristics such as age, education, gender, personality, experience and coping profiles, working conditions such as high or low job demands, irregular or long working hours, time pressure, job insecurity, lack of social support and psychological harassment, living conditions, and responses to stress. Therefore, the model consists of four main components: risks for work-related stress, individuals’ characteristics, stress reactions, and long-term consequences of stress. It defines the relationship within the components as a dynamic process. One of the most important advantages of the model is that it takes into account the individual differences of the workers. According to the model and various studies, short-term stress increases the motivation and productivity of workers but exposure to long-term stress resources causes various long-term health and behavioural problems. The model illustrates the long-term consequences of such stress reactions, which affect workers’ physical and mental health, job performance, work absenteeism, and other risky health behaviours. Generally, working conditions have a strong relationship with stress and its results (see Fig. 1) [6, 21–23]. Related studies have provided important evidence on the interrelations between the model’s components. For example, individual characteristics, such as age, education level, gender, goals, social support [24], and family conditions, have significant effects on one’s ability to cope with stress [25]. Moreover, working conditions, such as long working hours, lack of control over one’s workload, time pressures, job insecurity, and an insufficient salary, have a strong influence on the development of job-related stress [26].

It is widely acknowledged that perceived social support plays a significant role in decreasing WRS. It also has positive effects on job performance, work absenteeism, and productivity [24, 27–29]. The higher the degree of social support workers have, the more easily they can be protected from occupational stress as well as from physical and mental health problems [30]. According to the studies with controlled study designs, interventions, such as exercise, education, consultations, or organizational programmes initiated by supervisors and administrators encourage feelings of solidarity and happiness. These kinds of feelings increase the amount of social support over the course of time [31].

The effects of stress vary between genders, placing females in a more disadvantaged and vulnerable category than their male counterparts due to biological and psychosocial systems. Gender discrimination, income inequality, and cultural barriers play an important role on this matter, especially in the developing and undeveloped countries [2, 32–36]. Research on how stress affects different genders reveals that women are twice as likely as men to develop symptoms of stress. The identified underlying causes of this include the female biological and psychological systems, the impact of women’s many roles and responsibilities concerning family life, and their exposure to societal disparities. One’s cultural perspective also has a significant impact on preventing and coping with WRS [33, 37–39]. However, working conditions and the economy have presented employees with more difficulties in recent years. Second, only to child workers, women are the most vulnerable population of employees. It is, therefore, crucial to prevent job stress, as much as possible, before it causes chronic problems for all workers, especially women workers. Women workers in particular have disadvantaged life and working conditions due to high social disparity, gender inequality, and a great responsibility to balance work and family life in undeveloped and antisocial-democratic society. Accordingly, primary protective mental health intervention should be implemented at work; however, such assistance has been found to be insufficient, and pre-test and post-test control studies are lacking in developing countries [40].

As a result, various approaches relating to prevention, protection, and promotion programmes covering stress management have been introduced and implemented in various countries [17, 26, 41, 42]. However, a majority of intervention programmes are focused on limited specific topics (e.g. job stressor, psychological distress, sickness absenteeism, IgA) in developed countries. Therefore, this WRS model-based study was designed to plan and evaluate Workplace Mental Health Promotion Program (Work-ProMentH) using a broad, systematic approach to women workers’ health in a developing country. The WRS model enables its users to assess the causes and consequences of work-related health using a holistic approach and to plan and evaluate programmes in a systematic manner (see Fig. 1) [6]. Additionally, women workers were chosen as the specific research samples in the present study for the reasons mentioned above, and working in the textile and garment sectors is very common among the workers. This sector has the highest rate of women workers compared to other sectors in some countries such as Turkey. Generally, women who work in this sector have low levels of education, are unskilled and have poor economic status. Precarious working conditions such as job insecurity, unpredictable working hours, insufficient salary, and lack of a union are very common among the workers in this sector [43–45]. As a result, the aim of this study was to examine the effects of the newly developed WRS model-based Workplace Mental Health Promotion Program (Work-ProMentH) on the job-related stress of female workers, their physical and mental reactions to stress, social support, coping profiles, work absenteeism, and job performance; the women’s salivary immunoglobulin A (S-IgA) and cortisol (S-cortisol) levels were also measured.

## Methods

### Study design and objectives

The aim of this study, which featured a pre-test–post-test non-equivalent control group design, was to examine the effects of the WRS model-based Work-ProMentH on women workers’ job stress, physical and mental reactions, social support, coping profiles, work absenteeism, job performance, and salivary IgA (S-IgA) and cortisol (S-cortisol) levels. The research was carried out in 2 textile factories (A and B) since the intervention is considered to affect workers in the same factory. Factory B is defined as a subcomponent and partner of factory A. The factories mainly manufacture knitwear and export it abroad. Both factories have demonstrated adherence to the laws and regulations on occupational health and common international inspections. The factories also have the same occupational health physician and nurse. Additionally, the first researcher voluntarily worked 2 hours per week for more than a year before the study began to observe the work process, working conditions, and work environments.

### The hypotheses we tested in this study are as follows

Compared to those who do not participate in the Work-ProMentH intervention, workers who do participate in the program will have decreased job stress, less severe physical and mental stress reactions, lower S-cortisol levels, less job absenteeism, increased S-IgA levels, more social support, better job performance, and improved coping profiles.

### Participants

**Fig. 2:**
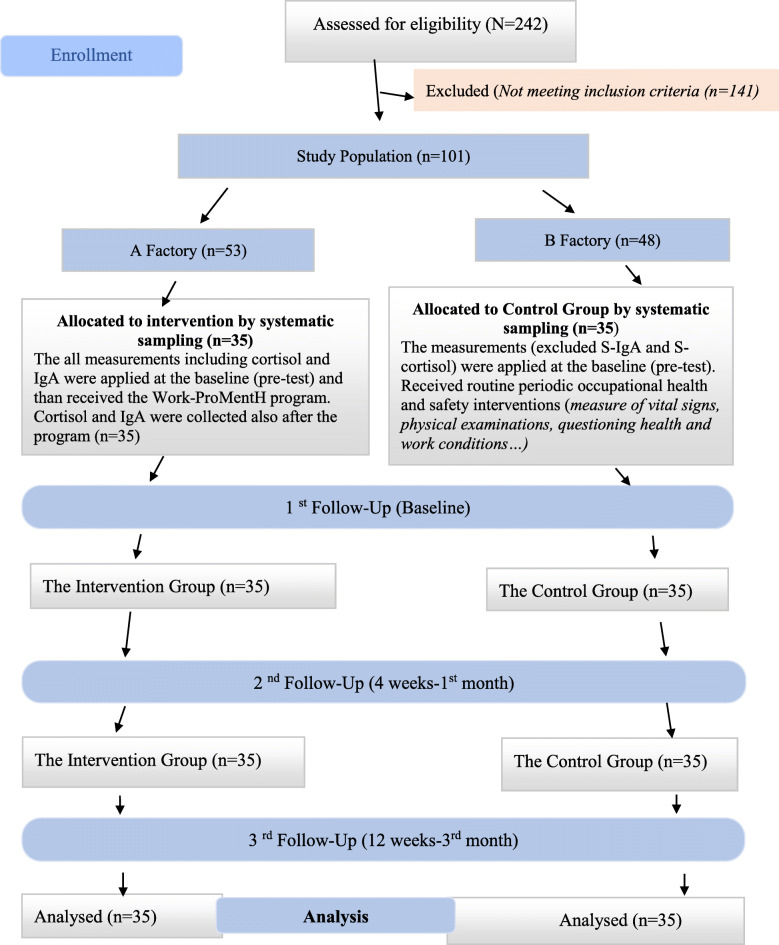
The Flow Diagram of the Study

Criteria for inclusion in the study sample consisted of a job-stress subscale score above the median(med: 45), indicating a higher level of WRS, and no use of any medication that has effects on cortisol and IgA. Criteria for exclusion from the study population included the use of any medication that affects salivary cortisol and/or IgA levels, age under 18 years, diagnosed psychiatric health problems, or illiteracy. Out of 242 female workers assessed for eligibility, 101 (53 from factory A, and 48 from factory B) met the criteria and were included in the study. The factory where the programme was performed was selected by a draw (factory A). The study sample size was calculated by power analysis; the minimal study sample was found to be 58 (intervention group [IG]: 29, control group [CG]: 29). The acceptance of Type I errors was set at 5%, and that of Type II errors was set at 20% (α = 0.05, 1-β = 0.80). Drop-outs were expected rate for unknown reasons. Researchers selected 70 participants (35 in each study group) from factories A and B via systematic sampling. The study procedure is shown in Fig. 2. All workers worked 5 days per week, beginning at 08:30 am, and finishing at 7:00 pm. Their lunch was provided by the factories. The mean age of the women workers in the IG was 33.54 ± 9.6 (19–54) years, and almost 49% of them graduated from elementary school. The mean age of the women workers in the CG was 31.11 ± 8.2 (20–52) years, and almost 60% of them graduated from elementary school. There was no difference between the groups’ main sociodemographic characteristics. The characteristics of the groups are reported in Table 1.

**Table 1:** Comparison of Sociodemographic Characteristics of the Intervention and Control Groups

Variables	Intervention(*n* = 35) n %	Control(*n* = 35) n %	Statistic	
Age ($\overline{\mathrm{x}}$ ±SD)	33.54 ± 9.6	31.11 ± 8.2	Z = -917	*p* = 0.359*
Marital status				
Married	18 (51.4)	14 (40)	x^2^ = 0.305	*p* = 0.581*
Single	17 (48.6)	21 (60)		
Having Children				
Evet	13 (37.2)	13 (37.2)	x^2^ = 0.015	*p* = 0.901*
No	22 (62.8)	22 (62.8)		
Education status				
Elementary school	17 (48,6)	21 (60)		
Primary school	4 (11.4)	10 (28.6)	x^2^ = 6.808	*p* = 0.339*
High school	10 (28.6)	4 (11.4)		
Baccalaureate	4 (11.4)	–		
Health Conditions				
Very good	–	3 (8.6)		
Good	30 (85.7)	27 (77.1)	x^2^ = 1.728	*p* = 0.421*
Bad	5 (14.3)	5 (14.3)		
Very bad	–	–		
To Having Chronic disease				
Yes	5 (14.7)	7 (20)	x^2^ = 0.000	*p* = 1.000*
No	30 (85.7)	28 (80)		
Economic status				
Very good	–	…….		
Good	30 (85.7)	6 (17.1)	x^2^ = 5.314	*p* = 0.070*
Bad	4 (11.4)	……..		
Very bad	1 (2.9)	29 (82.9)		
Working experience (year)	4,91 ± 3,84	5.09 ± 3.00	Z = -1.108	*p* = 0.268*
Salary/month (Turkish Lira)				
Between 800 and 1500	30 (85.7)	32 (91.4)		
Between 1501 and 2200	1 (2.9)	3 (8.6)	x^2^ = 11.181	*p* = 0.985*
Between 2201 and 2700	1 (2.9)	–		
2701 and over	3 (8.6)	–		
Age of beginning to work in life				
18 years old and younger	20 (57.2)	22 (62.8)		
19 years old and older	15 (42.8)	13 (37.2)	x^2^ = 1.020	*p* = 0.313*

*p>0.05 × ^2^ = Chi-square Z = Mann-Whitney U test

The data were collected between February and April, 2016. During this process, 2 participants (due to marriage) and 3 participants (due to working conditions and health problems) were dropped from the CG between weeks 10 and 11 of the follow-up stage. The workers who dropped out were not considered missing as the intent-to-treat (ITT) principle was used in the data analysis process [46].

### Measurements

The study examined five measurements that were selected and developed by researchers based on the components (Risks for work-related stress, Stress reactions, Stress long-term consequences, Individuals characteristics) of the causes and consequences in the WRS model to evaluate stress, stress reactions, long-term stress responses, and coping profiles. The risks for the work-related stress component of the model were measured by the brief job stress questionnaire and the assessment form; stress reactions were measured by the brief job stress questionnaire and salivary-specific ELISA kits; individual characteristics were measured by the Brief Stress Coping Profile scale and the assessment form; and stress long-term consequences were measured by the performance questionnaire, formal, digital factory input and output card and checklist (see Fig. 1).

I. The Descriptive Workers Assessment Form concerns sociodemographic characteristics (age, gender, education, birthplace, marital status, perception of economic condition, and classification of salary per month); health characteristics (perceptions about the participant’s health, quality of sleep, and any chronic disease(s) being medically controlled or treated); work characteristics (work hours, occupation, work schedule, and frequency and duration of work breaks and annual leave); and perceived job performance questions that were developed by the World Health Organization. Responses to the questions vary from 1 to 10 points, with 1 point representing the lowest job performance and 10 points representing the highest job performance [47].
II. The Brief Job Stress Questionnaire (BJSQ), a self-assessment form developed in Japan [48], consists of 57 items covering job stress (17 items such as “I have an extremely large amount of work to do”, “I can’t complete work in the required time”, “The atmosphere in my workplace is friendly”), physical (11 items such as “I have experienced stomach and/or intestine problems”, “I have experienced diarrhoea and/or constipation”, “I have had a stiff neck and/or shoulders”, “I have had lower back pain”) and psychological stress reactions (18 items such as “I have been inwardly annoyed or aggravated”, “I have felt tense”, “I have felt gloomy”), and social support *(11 items)* [49]. It has a 4-point Likert-type response option from “strongly disagree” =1 to “strongly disagree” =4. A higher subscale score indicates a high level of job stress, stress reactions, or social support [50]. The Turkish version of the BJSQ was used in this study, and the scale’s reliability and validity were measured before conducting the research. The reliability of the Turkish version of the BJSQ’s subscales was indicated by Cronbach’s alpha values of 0.66, 0.81, 0.82, and 0.81, respectively [51]. Additionally, the Cronbach’s alpha values of these subscales were found to be between 0.66 and 0.90 in this study. The BJSQ was used at the baseline and follow-up stages.
III. The Brief Stress Coping Profile (BSCP), comprising 18 related items on a 4-point Likert-type scale (often, sometimes, seldom, or never), is a self-rating scale for assessing workers’ coping profiles. The questionnaire was developed by Kageyama, Kobayashi, Kawashima, and Kanamaru (2004) [52] and features the following six subscales: active solution (items 1, 2, and 3; such as “I try to analyse the causes and solve the problem”), seeking help for the solution (items 4, 5, and 6; such as “I consult with someone I can trust”), changing mood (items 7, 8, and 9; such as “I try to do something that calms me down”), changing one’s point of view (items 10, 11, and 12; such as “I try to think this experience is good for me”), emotional expression involving others (items 13, 14, and 15; such as “I blame the person who is involved in the problem”), and avoidance and suppression (items 16, 17, and 18; such as “I do nothing but endure the situation”). Each of these subscales has 3 items and a score range of 3–12 points. A high subscale score indicates that the respondent frequently chooses that kind of coping method [53]. The Turkish version of the BSCP was used in this study, and the scale’s reliability and validity were measured before conducting the research. The reliability of the Turkish version of the BSCP were indicated by Cronbach’s alpha values of 0.69, 0.71, 0.66, 0.75, 0.78, and 0.77, respectively [54]. The BSCP was used at the baseline and follow-up stages.
IV. Salivary-specific ELISA kits that are lucent and have a cover were used to evaluate cortisol and IgA levels in every participant’s saliva. When saliva is collected with the kit, it should be covered carefully and saved in a portable freeze at + 4°, and it has to be transferred to a laboratory with an International Accreditation. The analyses were conducted at baseline (at 08:45 am, February) and just after the IG intervention (at 10:15 am, February) in the morning. The eligibility criteria for collecting saliva-cortisol and saliva-IgA are as follows: there should not be any blood contamination from the mouth, there should not be any medication that affects cortisol used in the last week, and there should not be anything eaten 30 min before saliva collection.
V. The work absenteeism of all participants was checked through formal digital factory timecards and self-reported checklists. The absenteeism duration was calculated based on hours.

### Workplace mental health promotion program intervention

The Work-ProMentH is a health-promotion programme based on the WRS model [6]. Before the Work-ProMentH intervention, the approval and follow-up procedures of the factories’ administration were explained to the IG and verified. The programme was applied once to the IG (35 women workers) at baseline in factory A at 9 am by the first researcher. A visual presentation and video training were provided, and a digital camera was used to video record workers while they were practising the programme, which consisted of stress management techniques, effective coping skills, and relaxation exercises. The programme also provided definitions for and explanations of stress and WRS, stress physiology, stress reactions, stress-related diseases, stress-related factors and effective coping and stress management skills, relaxation exercises, and deep breathing techniques. In the context of coping with stress, these exercises were taught, along with correct abdominal deep breathing skills, to the IG. During this interactive training, the WRS factors were defined and discussed interactively and in detail with the workers, who offered examples from their work experiences. During the training, the exercises were practised as a group and video recorded. The training lesson lasted 45 min and took place in a meeting room at the workplace. After the training, a brochure explaining the content and process of the programme and a video describing the exercises were given to the workers. The exercise times (10:00 a.m., 1:00 p.m., and 4:00 p.m.) were organized in cooperation with the workers, supervisors, and administrators while considering the employees’ work schedules. This was followed by direct observation, a weekly self-reported checklist, and recording via a factory-fixed camera for 12 weeks. The mobile phone application WhatsApp was used to send reminder messages and videos to the participants to reinforce the training during the follow-up stage. The effects of the programme were assessed in the first and third months in both groups, but S-IgA and S-cortisol levels were assessed only in the IG before and just after the intervention (see Fig. 2).

### Statistical analysis

Statistical analyses were conducted using SPSS version 22 for Windows (SPSS, Inc., Chicago, IL, USA). Descriptive statistics of the demographic characteristics and health and work conditions of the workers are presented as numbers, percentages, and means ± standard deviations. A chi-square test and Mann-Whitney U test were applied for comparing the groups’ sociodemographic characteristics. An independent samples t test was performed to analyse the difference between the means of the groups, and a paired sample t test was conducted to analyse the difference between the pre-post mean scores of the variables. Repeated measures ANOVA was performed to detect the difference between related means of each group by time. The Bonferroni test was conducted to correct for multiple testing. Partial eta squared (η_p_^2^) was then used for the overall effect. Using Cohen’s guidelines (1988), η_p_^2^ = .01 was considered small, η_p_^2^ = .06 was considered moderate, and η_p_^2^ = .14 was considered a large effect [55]. Data were evaluated with a 95% confidence interval, and *p* < 0.05 was accepted as significant.

## Results

### Primary outcomes

The sociodemographic characteristics, general health, working conditions, job stress, stress reactions, social support, job performance, work absenteeism, and coping profiles of the female workers were evaluated and compared before the Work-ProMentH was initiated. In addition, the IG’s S-IgA and S-cortisol levels were assessed prior to the programme’s start.

### Demographic characteristics, general health, and work conditions

The mean age of the workers was 32.3 years (SD = 9.01, with a range of 19–54); more than 54% (*n* = 38) had completed primary education, almost 46% of them were married, and over 62% of them had no children. Of all participants, 81.4% reported their health condition as “good,” and over 77% of those who reported their health condition as “good” were among the CG. All participants worked 5 days per week, 12 h per day, and took their breaks at the same time. None of them had permanent working contracts or were members of unions. Over 51% of them defined their economic condition as “good,” and almost 86% of those who defined their economic condition as “good” were among the IG. Over 88% of them worked for a minimum wage (800–1500 Turkish Lira = 510–748 U.S. dollars) in the factories. More than 57% of the IG and almost 63% of the CG began to work when they were younger than 18 years old. The mean working experience was 4.91 years (SD = 3.84) in the IG and 5.09 years (SD = 3.00) in the CG. A comparison of women workers in the IG and CG revealed no differences in sociodemographic characteristics, general health condition, work experience, or working conditions (*p* > .05) (see Table 1).

### Job stress, stress reactions, social support, job performance, work absenteeism, and coping profiles

The mean scores for job stress, physical symptoms (e.g., stomach, back, or arm pain), mental stress reactions (e.g., depression, irritability, annoyed mood), social support, job performance (hours/month), work absenteeism (hours/month), and coping profile in the IG and CG were compared. There were no differences between the IG and CG in terms of the scores for these variables (*p* > .05) (see Table 2).

**Table 2:** The comparison of Job Stress, Stress Reactions, Social Support, Job performance, Work absenteeism, S-IgA, S-Cortisol and Relaxation Exercises’ Mean Values (Intervention/Control: *N* = 35)

Components	Groups	Before Work ProMentH (Baseline) ($\overline{\mathrm{x}}$ ±Sd)	After Work-ProMentH 1st month ($\overline{\mathrm{x}}$ ±Sd)	After Work-ProMentH 3rd month ($\overline{\mathrm{x}}$ ±Sd)	Statistic
Job stress	Intervention Difference within time	47.91 ± 5.81	40.60 ± 4.48	38.25 ± 4.13	F = 113.99 df = 2 *p* = .000 1–2-3
	Control	45.57 ± 4.23	46.00 ± 5.45	46.57 ± 4.52	F = .523 df = 2 *p* = .595
	Statistic	t^a^ = 1.928 *p* = .058	t^a^ = −4.523 *p* = .000	t^a^ = −8.019 *p* = .000	
Mental reactions	Intervention Difference within time	42.37 ± 7.17	37.14 ± 7.68	37.71 ± 8.45	F = 7.947 df = 2 *p* = .001 1–2;1–3
	Control	38.40 ± 10.75	39.20 ± 8.26	39.14 ± 8.62	F = .202 df = 2 *p* = .818
	Statistic	t^a^ = 1.816 *p* = .074	t^a^ = −1.078 *p* = .285	t^a^ = −.699 *p* = .487	
Physical reactions	Intervention Difference within time	24.02 ± 5.10	20.97 ± 4.72	19.25 ± 4.48	F = 33.444 df = 2 *p* = .000 1–2-3
	Control	23.02 ± 5.10	23.11 ± 5.82	22.40 ± 4.80	F = .570 df = 2 *p* = .568
	Statistic	t^a^ = .780 *p* = .438	t^a^ = −1.689 *p* = .096	t^a^ = −2.829 *p* = .006	
Social support	Intervention Difference within time	30.17 ± 4.71	34.94 ± 4.12	37.37 ± 3.42	F = 56.342 df = 2 *p* = .000 1–2-3
	Control	32.37 ± 4.88	32.85 ± 4.86	32.85 ± 5.35	F = .254 df = 2 *p* = .776
	Statistic	t^a^ = −1.917 *p* = .059	t^a^ = 1.935 *p* = .057	t^a^ = 4.204 *p* = .000	
Perceived job performance	Intervention Difference within time	8.08 ± 2.03	8.48 ± 1.33	9.08 ± 1.06	F = 4.701 df = 2 *p* = .021 1–3;2–3
	Control	8.74 ± 1.33	8.40 ± 1.51	8.37 ± 1.26	F = .944 df = 2 *p* = .394
	Statistic	t^a^ = −1.597 *p* = .115	t^a^ = .251 *p* = .803	t^a^ = 2.556 *p* = .013	
Work absenteeism (hours/month)	Intervention Difference within time	12.85 ± 11.00	11.17 ± 9.82	7.65 ± 8.17	F = 3.735 df = 2 *p* = .029 2–3
	Control	13.88 ± 16.80	9.82 ± 10.83	13.22 ± 13.49	F = 2.986 df = 2 *p* = .057
	Statistic	t^a^ = −.303 *p* = .763	t^a^ = .543 *p* = .589	t^a^ = −2.089 *p* = .040	
		After Work-ProMentH 1st month ($\overline{\mathrm{x}}$ ±Sd)		AfterWork-ProMentH 3rd month ($\overline{\mathrm{x}}$ ±Sd)	
Relaxation Exercises	Intervention	71.60 ± 33.39		129.45 ± 78.86	t^b^ = −6.611 *p* = .000
		Before Work-ProMentH ($\overline{\mathrm{x}}$ ±Sd)		After Work-ProMentH ($\overline{\mathrm{x}}$ ±Sd)	
S-IgA (ug/ml)	Intervention	110.32 ± 88.37		83.67 ± 68.45	t^b^ = 2.242 *p* = 0.03
S-cortisol (ng/ml)	Intervention	5.60 ± 1.53		4.12 ± 1.27	t^b^ = 5.302 *p* = 0.000

^a^ = Independent Samples Test ^b^ = Paired Samples Test F = repeated measures ANOVA/Sphericity assumed or Green house-Geisser test df = degree of freedom

### S-IgA and S-cortisol scores

S-IgA and S-cortisol levels were analysed in the IG. The mean S-IgA score was 110.32 ± 88.37, and the mean S-cortisol score was 83.67 ± 68.45 (see Table 2).

### Secondary outcomes

#### Work-ProMentH intervention follow-up

The IG received the Work-ProMentH with follow-up for a 3-month period. Female workers in the IG and CG were compared at baseline and in the first and third months with respect to the frequency of relaxation exercises, job stress, physical and mental reactions, S-IgA and S-cortisol levels, social support, coping profiles, job performance, and work absenteeism (see Table 1).

According to the findings at the follow-up, the mean scores in the IG for practising the relaxation exercises were 71.60 (SD = 33.39) in the first month and 129.45 (SD = 78.86) at the end of the third month. There was a significant increase in the mean scores for the groups’ performance of relaxation exercises (*p* ≤ .001). During the first month, 62.9% (*n* = 22) regularly implemented the programme compared with 51.4% (*n* = 18) at the end of 3 months. At the end of the first month, the reasons for not implementing the intervention regularly were reported as follows: 75% “*had a heavy workload,*” 15.5% *“felt sick,”* 4.5% *“were not able to concentrate,”* and 5% *“did not feel like doing it.”* By the end of the third month, the reason “*did not feel like doing it*” increased to 17%, and “*felt sick*” decreased to 5%.

#### Intervention effects on job stress, stress reactions, social support, S-IgA and S-cortisol levels, job performance, and work absenteeism

According to the present findings from the Work-ProMentH follow-up, workers in the IG reported decreased job stress (*p* ≤ .001; η_p_^2^ = .77) and mental (*p* = .001, η_p_^2^ = .18) and physical (*p* ≤ .001; η_p_^2^ = .49) reactions. Scores on post-test follow-up 1 and 2 were significantly lower than the pre-test scores. There was also a significant increase in the IG workers’ perceived job performance (*p* = .02; η_p_^2^ = .12) and social support (*p* ≤ .001; η_p_^2^ = .62) and a significant decrease in their work absenteeism (*p* = .029; η_p_^2^ = .09). In terms of subjective job stress, IG post-test scores were significantly reduced compared with the pre-test scores; however, the CG post-test job-stress scores were slightly increased. A comparison of the IG and CG mean scores for all variables noted here before and after the Work-ProMentH follow-up revealed a significant difference in the third months’ results, except for those for mental reactions (*p* = .487). A majority of effect sizes were very large (η_p_^2^ > .14 was considered large).

The IG S-IgA enzyme and S-cortisol hormone levels were analysed before and after Work-ProMentH and showed statistically significant reductions (*p* = .03 and *p* ≤ .001, respectively).

#### Intervention effects on coping profile

Overall, a significant effect of Work-ProMentH on coping profile was found. Table 3 shows a comparison of the coping profile mean values at the follow-up. There was a significant increase in the coping profile mean scores for active solution (*p* = .014; η_p_^2^ = .11), seeking help for the solution (*p* = .032; η_p_^2^ = .10), changing mood *(p* = .007; η_p_^2^ = .13) and changing one’s point of view (*p* = .004; η_p_^2^ = .17); however, there were decreases in coping profile scores for emotional expression involving others (*p* ≤ .001; η_p_^2^ = .33) and avoidance and suppression (*p* = .004; η_p_^2^ = .21) at follow-up. According to these results, the profiles of the workers showed significant improvements at follow-up; however, a comparison of the IG and CG revealed that the programme did not demonstrate any statistically significant differences in active solution profiles.

**Table 3:** The comparison of the Brief Stress Coping Profiles’ Mean Values (Intervention/Control: *N* = 35)

Coping Profiles	Groups	Before Work ProMentH (Baseline) ($\overline{\mathrm{x}}$ ±Sd)	After Work-ProMentH 1st month ($\overline{\mathrm{x}}$ ±Sd)	After Work-ProMentH 3rd month ($\overline{\mathrm{x}}$ ±Sd)	Statistic
Active solution	Intervention	8.97 ± 2.09	9.85 ± 1.59	9.94 ± 1.51	F = 4.577 df = 2 *p* = .014
	Control	9.62 ± 1.69	9.65 ± 1.55	9.40 ± 1.26	F = .526 *p* = .594
	Statistic	t^a^ = −1.442 *p* = .154	t^a^ = .532 *p* = .596	t^a^ = 1.628 *p* = .108	
Seeking help for solution	Intervention Difference within time	9.65 ± 2.07	10.54 ± 1.35	10.42 ± 1.37	F = 3.915 df = 2 *p* = .032 1–2
	Control	9.14 ± 1.84	8.57 ± 1.50	9.02 ± 1.63	F = 1.325 *p* = .273
	Statistic	t^a^ = 1.096 *p* = .277	t^a^ = 5.763 *p* = .000	t^a^ = 3.872 *p* = .000	
Changing mood	Intervention Difference within time	8.71 ± 2.51	9.80 ± 1.34	9.88 ± 1.45	F = 5.300 df = 2 *p* = .007 1–3
	Control	8.85 ± 2.11	8.74 ± 1.33	8.28 ± 1.60	F = 1.072 *p* = .348
	Statistic	t^a^ = −.257 *p* = .798	t^a^ = 3.298 *p* = .002	t^a^ = 4.382 *p* = .000	
Changing a point of view	Intervention Difference within time	9.82 ± 1.90	10.54 ± 1.09	10.71 ± 0.89	F = 6.983 df = 2 *p* = .004 1–2.1–3
	Control	10.00 ± 1.59	9.74 ± 1.73	9.31 ± 1.69	F = 2.575 *p* = .088
	Statistic	t^a^ = .798 *p* = .684	t^a^ = 2.305 *p* = .024	t^a^ = 4.325 *p* = .000	
Emotional expression involving others	Intervention Difference within time	7.80 ± 2.11	6.62 ± 1.53	5.34 ± 1.67	F = 16.830 df = 2 *p* = .000 1–2-3
	Control	7.40 ± 2.13	7.51 ± 2.00	7.42 ± 1.86	F = .032 *p* = .968
	Statistic	t^a^ = .789 *p* = .433	t^a^ = −2.075 *p* = .042	t^a^ = −4.913 *p* = .000	
Avoidance and suppression	Intervention Difference within time	7.14 ± 2.19	7.14 ± 2.19	5.94 ± 1.13	F = 9.541 df = 2 *p* = .004 1–3. 2–3
	Control	8.05 ± 2.37	8.02 ± 1.85	8.22 ± 1.45	F = .191 *p* = .827
	Statistic	t^a^ = −1.671 *p* = .099	t^a^ = −1.822 *p* = .073	t^a^ = −7.319 *p* = .000	

^a^ = Independent Samples Test F = repeated measures ANOVA/Sphericity assumed test or Green house-Geisser

## Discussion

The comprehensive evidence-based results of this pre-test and post-test-controlled study included a 12-week (3 months) follow up. Randomized sampling was used to select the study population, but the study was carried out in 2 textile factories since the intervention is considered to affect workers in the same factory. That may be a limitation of the study. The Work-ProMentH’s effects on the job stress, physical and psychological stress reactions, social support, S-cortisol and S-IgA levels, job performance, work absenteeism, and coping profile of female workers were assessed. In this process, the WRS model was used as a guide to evaluate the dynamic relations of these factors. The results of the present study show that the Work-ProMentH had meaningful effects on the job stress, physical and mental stress reactions, social support, S-cortisol levels, job performance, work absenteeism, and coping profiles of the IG participants. It was seen that the more the Work-ProMentH was practised, the greater its positive effects on the IG. However, when all these variables were compared between the IG and CG at follow-up, it was observed that the programme did not make any statistically significant differences in “mental reactions” and “active coping profiles.” An unexpected finding was that S-IgA levels were decreased after programme implementation.

We also considered worker attendance in the programme’s follow-up stage. It was found that the frequency of continuing the Work-ProMentH in the workplace decreased during the third month compared with that of the first month, although the performance rate was still high. The reasons for not fully participating (3 times per workday) in the programme were questioned. The most frequently self-reported reason was “heavy workload,” but the rate of the reason “feeling sick” decreased over time. This could be explained by the positive influence of the programme on coping profiles. Generally, the workers found it easy to complete the whole programme while they were at the workplace, except the first week. We think that positive reactions and feedback by the administrators/supervisors also played an important role in the results of the present study. Additionally, practising the programme at work was possible, which might also have been convenient. Even though it was the factories’ first experience with implementing a promotional programme at work, which was unusual for administrators, supervisors, and workers, the overall adoption of the programme was substantial. Beyond that, the first researcher’s work experience in this workplace afforded better communication and relationships between the researcher and the factories’ staff. This experience may have contributed to the success of the programme. However, this study was conducted between February and April when there is generally not a high workload at the factories; it might have been more difficult to obtain these results in summer.

The results of the present study indicate that the Work-ProMentH is an important programme in terms of protecting workers from job stress and physical stress reactions. In the study, the programme also increased the social support and general work performance of the IG. Therefore, the hypotheses (*Workers who participate in the Work-ProMentH intervention will have decreased job stress, less severe physical symptoms, increased social support, and better job performance*) have been accepted. Moreover, the results of the study by Atlantis et al. (2004) supported this hypothesis. It was revealed that the stress, mental health, and physical function levels of the workers who received the exercise and behaviour modification programme were significantly decreased compared with those of the waiting-list group [11]. However, compared to those in the present study, the participants in that study consisted of male and female workers, some of whom performed shift work, and they were followed up for a longer time. On the other hand, there was no information about working hours and work type in that study, which are mainly accepted as common risk factors for WRS. Additionally, an intervention programme that consisted of aerobic exercise was found to be effective in women workers from various industries who had similar working hours/week to the textile women workers in the present study [56]. Beyond that, a Tai Chi intervention programme was implemented twice a week for 12 weeks to men working at ambulatory clinics who are probably more advantaged than textile women workers. Additionally, compared to the present study, in that study, the age group was older (66–71 years), which may have affected the results, but the study revealed that Tai Chi exercise had a meaningful influence on social support and stress management [31]. As seen from the example, intervention programmes with exercise have generally been found to be effective for WRS, its causes and outcomes regardless of participant sociodemographic characteristics.

The present study found no statistically significant differences between the IG and CG mental reactions. The hypothesis (*Workers who participate in the Work-ProMentH intervention will have decreased mental stress reactions*) has therefore been rejected. Mental reactions are important symptoms of WRS, but it takes time to recover from them [57]. The present study results showed that the mental reaction score at the third follow-up (end of the third month) was lower than that at the first month, indicating that this programme had significant effects on the reactions of the IG but did not result in statistically significant differences between the groups. Longer follow-up times may be necessary to see changes in mental health. Compared to WRS-related physical reactions, internal mental health improvements take longer to recover. General work absenteeism and job performance are long-term consequences of WRS, according to the model used in this study. These two components vary depending on the level of job stress and its reactions [58, 59]. In the present study, there were meaningful differences between the work absenteeism rate of the IG and that of the CG at both post-Work-ProMentH follow-ups. The IG’s average work absenteeism score decreased, while the CG’s average score increased. For example, just three workers from the CG left their jobs due to working conditions during the third month, which could be related to the job stress level in the present study. Therefore, the hypothesis about work absenteeism has been accepted.

Cortisol hormone and IgA are important biological reactions to stress, and their levels vary according to the level of stress. For example, S-cortisol levels increase in parallel to stress. Ongoing high cortisol levels have long-term negative effects on the body’s immune system [9, 10]. This study indicated that the Work-ProMentH had a significant effect on reducing S-cortisol levels. Therefore, the hypothesis “*Workers who participate in the Work-ProMentH intervention will have lower S-cortisol levels*” was accepted. Furthermore, it is known that cortisol concentration is influenced by circadian rhythm [60]. In the present study, the wake-up time of the workers on the cortisol collection day was not taken into consideration. It is unlikely for cortisol levels to have been influenced by the wake-up time in the present study; however, it should be noted in future studies. However, in the present study, the mean post-intervention S-IgA score of the IG was found to be meaningfully higher than that at baseline, though it was expected to be lower. S-IgA levels usually increase under acute stress as a means of coping and decrease as the stressed individual becomes relaxed [61, 62]; in the present study, S-IgA levels were decreased after the intervention. These decreases likely may have occurred because stress, stress reactions, and work-related causes were discussed with the workers during the intervention session. The workers may have felt stress when they were expressing themselves or because the programme and process were new for them, which could explain the increased S-IgA levels. However, further long-term research is needed. In contrast to this study, research by Pawlow and Jones (2005) and Taniguchi et al. (2007) showed increased IgA levels after short relaxation exercises were initiated among female workers and students [63, 64]. On the other hand, no significant effects on IgA levels related to exercise were found in a study conducted by Berger and O’Brien (1998) [65].

Effective coping strategies and skills play an important role in stress, and it is crucial for female workers who are trying to balance work and family. The Work-ProMentH was found to be effective in improving “seeking help for a solution,” “changing mood,” “changing one’s point of view,” “emotional expression involving others,” “active solution seeking”, and “avoidance and suppression” coping profiles among the workers. The hypothesis therefore was accepted. Working conditions, national worker rights, self-confidence, culture, and other socio-economic conditions may have effects on these coping profiles.

### Limitations

Randomized sampling was used to select the study population, but the study was carried out in 2 textile factories since the intervention is considered to affect workers in the same factory. Although there was no difference between the characteristics of the two factories, a “pre-test post-test design for non-equivalent control groups” design was chosen instead of an experimental design. Additionally, the effects of the intervention programme in this study were evaluated for only 3 months.

## Conclusion

The effect of the WRS model-based Work-ProMentH on job performance, social support, job stress, stress reactions, and work absenteeism was assessed in this study using a randomized, controlled pre-test and post-test design in stressed women workers. It was found that women workers who participated in the Work-ProMentH experienced a decreased prevalence of job stress, physical and mental stress reactions, work absenteeism, and S-cortisol levels, increased levels of social support and job performance, and improved coping profiles.

The effects of the intervention programme in this study were evaluated for only 3 months. Therefore, we suggest that researchers apply for programmes that will enable them to collect follow-up data for a longer period. Additionally, the factories were privately held, and all workers had non-permanent contracts and no union. We think that these precarious working conditions may make them feel insecure and stressed. Additionally, work-family conflicts are an important dimension among women workers, particularly in antisocial-democratic society. Therefore, we also suggest that researchers take into consideration the effect of these precarious working conditions and work-family conflicts on women workers’ mental health in upcoming studies. As a result, there is a need for more empirical studies that may support the data of the present study, but it is thought that the intervention can be maintained for the long term. We recommend that occupational health professionals at workplaces consider using this model-based cost-effective intervention, which is easy and practical to apply in real-life situations.

## Data Availability

The datasets used and/or analysed during the current study are available from the corresponding author on reasonable request.

## Abbreviations

WRS: Work-related stress
S-IgA: Salivary-Immunoglobulin A
Work-ProMentH: Workplace Mental Health Promotion Program
BSCP: The Brief Stress Coping Profile
BJSQ: The Brief Job Stress Questionnaire
